# Supplementary material for: Chemical Variability and Chemotype Concept of Essential Oils from Algerian Wild Plants
Source: Molecules. 2023 May 30;28(11):4439. doi: 10.3390/molecules28114439 (PMC10254628; doi:10.3390/molecules28114439)
Supplement: Supplementary file 1 [file molecules-28-04439-s001.zip › molecules-2343353-supplementary.pdf]

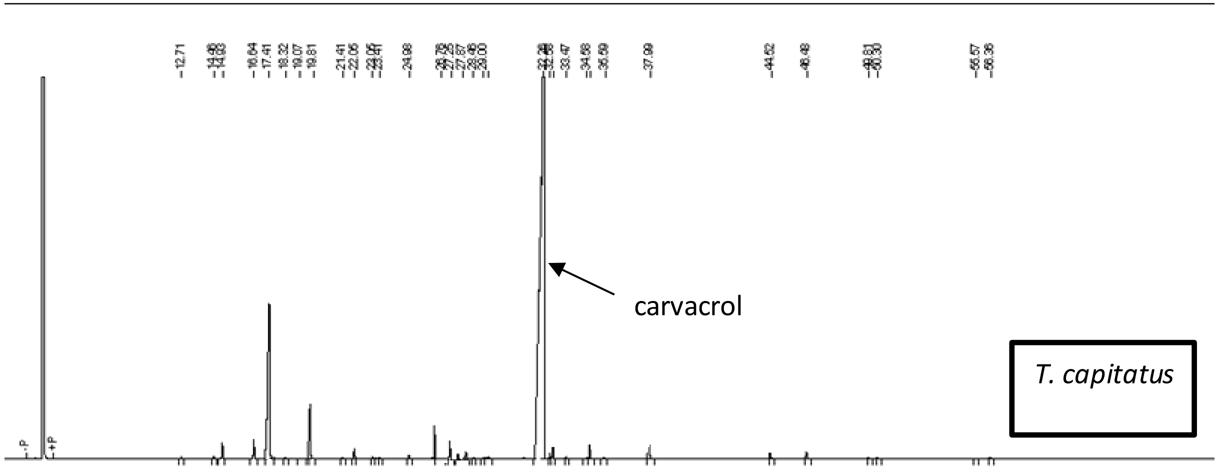

Figure S1. Essential oil chromatogram of *Thymus capitatus*

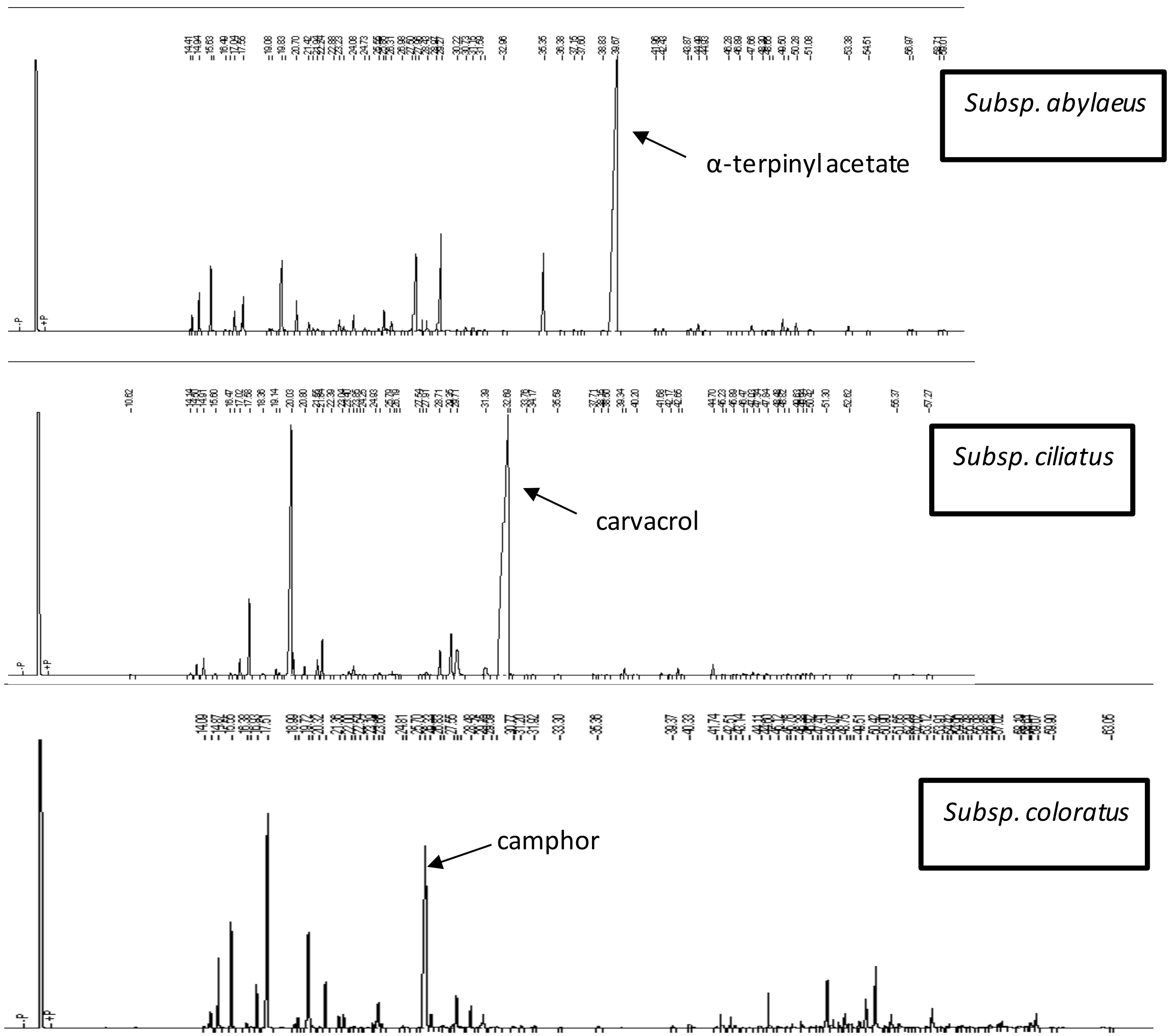

Figure S2. Essential oil chromatogram of *Thymus munbyanus* subspecies

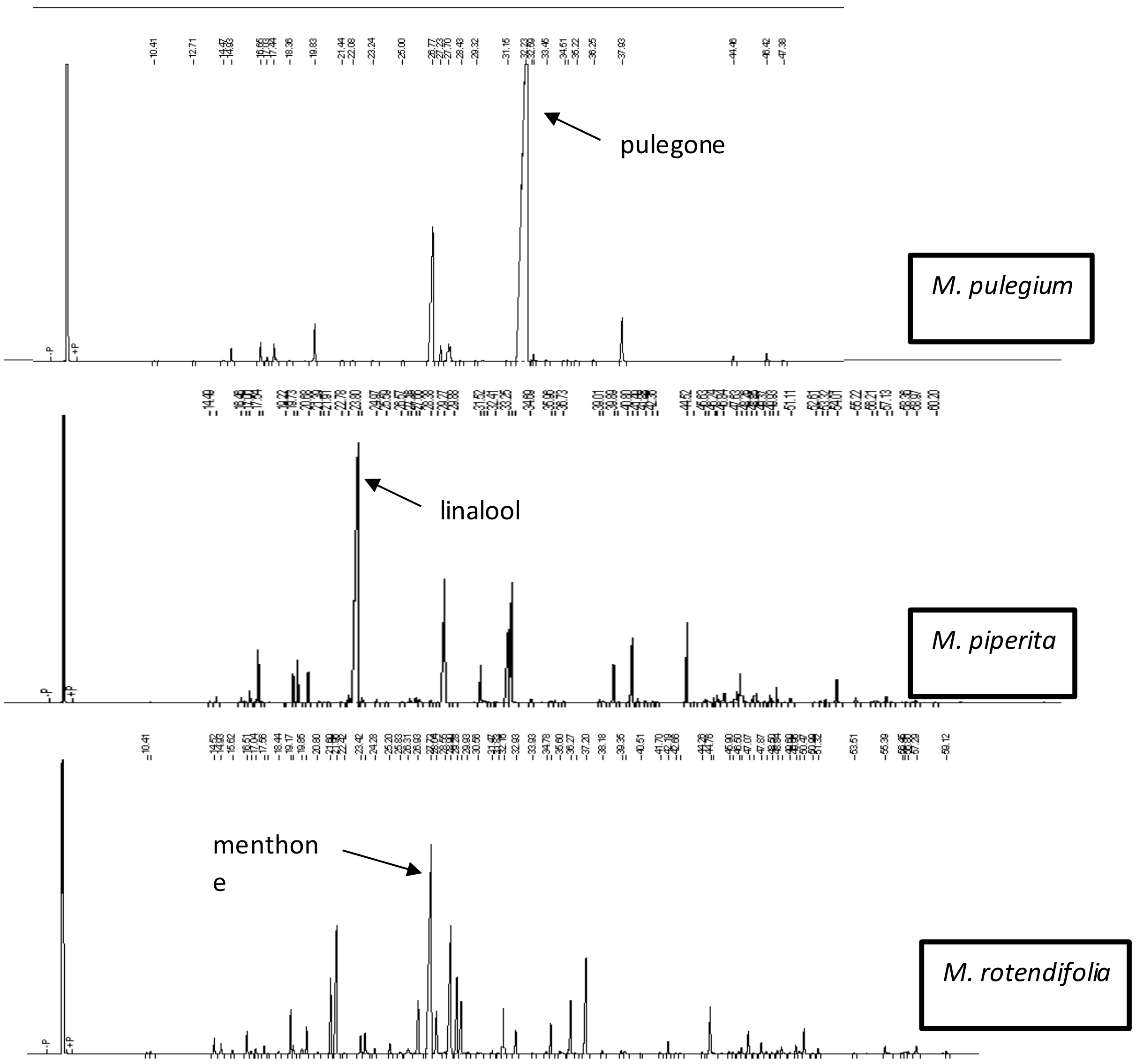

Figure S3. Essential oil chromatogram of *Mentha* species

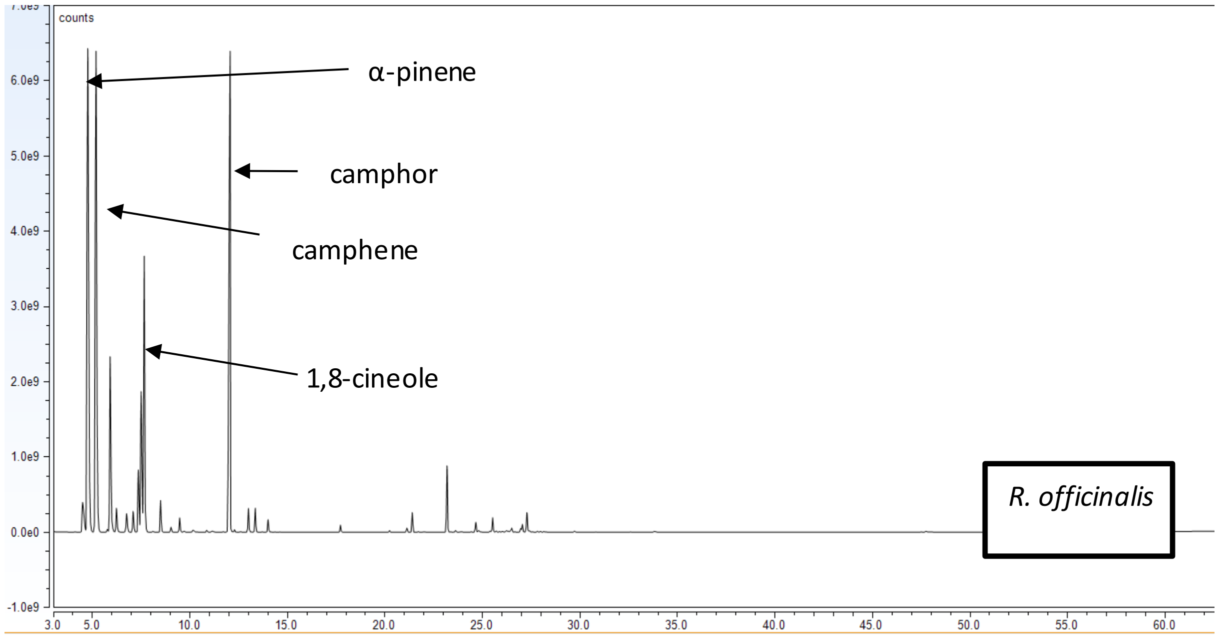

Figure S4. Essential oil chromatogram of *Rosmarinus officinalis*

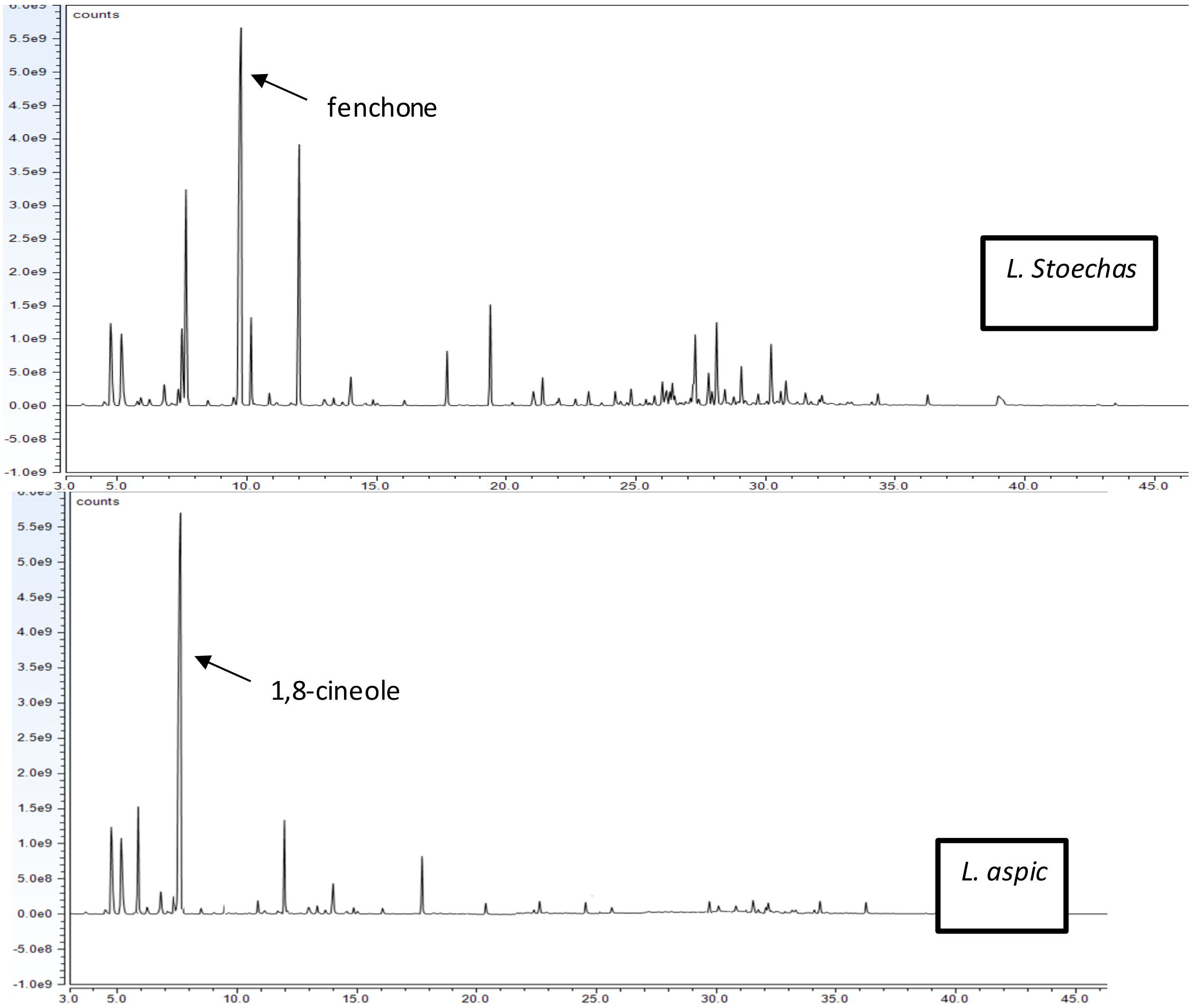

Figure S5. Essential oil chromatogram of *Lavandula* species

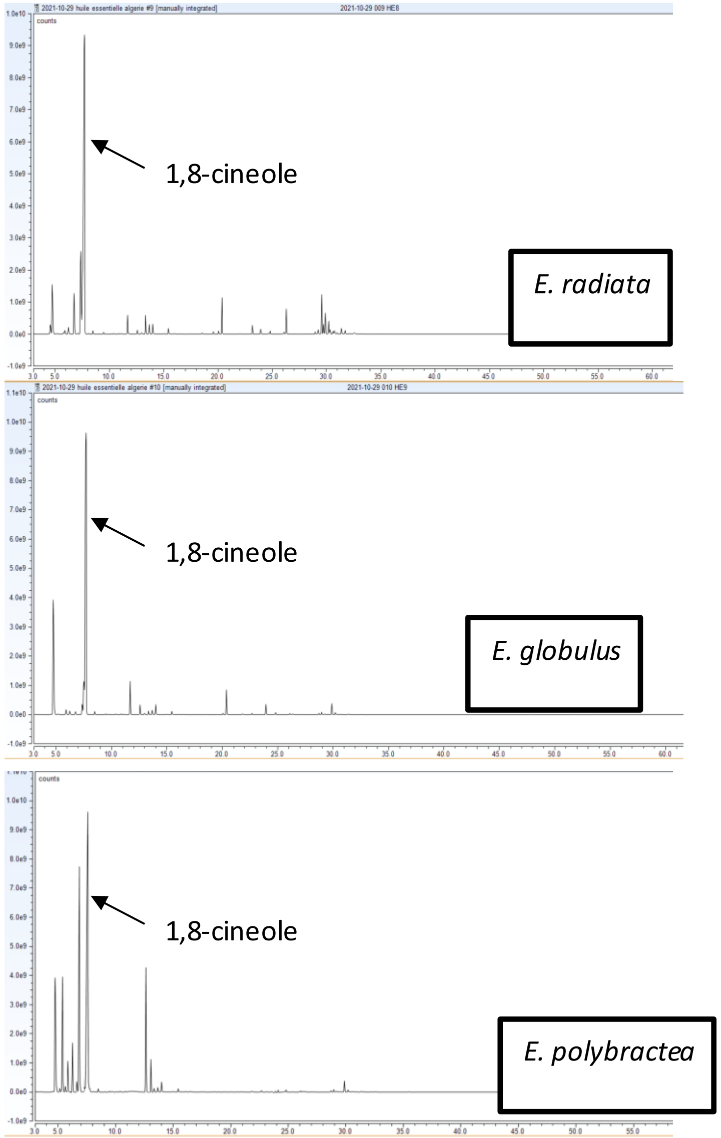

Figure S6. Essential oil chromatogram of *Eucalyptus* species

Table S1. Chemical composition of essential oils of different species.

No	Compound name and classa[a]	lRla[b]	RIa[c]	RIp[d]	*Thymus*	*Thymus munbyanus* subsp.[e]			*Mentha*			*Rosmarinus*		*Lavandula*		*Eucalyptus*			Identification[f]
					capitatus	*abylaeus*	*ciliatus*	*coloratus*	*pulegium*	*piperita*	*rotundifulia*	*officinalis*		*stoechas*	*aspic*	*radiata*	*globulus*	*polybractea*	
1	(E)-Hex-3-en-1-ol	812	810	1360							tr								RI, MS
2	Ethyl-2-methyl butyrate	829	829	1016							0.1								RI, MS
3	(E)-2-Hexenal	830	830	1210						0.1	0.1								RI, MS
4	(Z)-Hex-3-en-1-ol	831	832	1375							0.1								RI, MS
5	(Z)-2-Hexenol	851	848	1400							tr								RI, MS
6	1-Hexenol	852	851	1414							tr								RI, MS
7	tricyclene	921	923	1020		0.5						0.1	tr	0.1					RI, MS
8	α-thujene	922	925	1023	0.2	0.3	0.6	0.8	0.1	tr	0.2	0.1	tr			0.1			RI, MS
9	α-pinene	931	933	1022	0.9	1.6	0.6	3.1	0.5	0.2	0.4	6.8	24.4	1.7	3	5.4	6.3	4.5	RI, MS
10	camphene	943	946	1066	0.2	3.2	0.1	6.2			tr	3.4	21.5	2.1	0.1	0.5			RI, MS
11	thuja-2,4(10)diene	946	947	1120		0.1						0.1	tr	0.2					RI, MS
12	oct-1-en-3-ol	959	956	1446	0.5	0.1		0.3	0.8		0.5	0.2	tr						RI, MS
13	sabinene	964	962	1120		0.1	0.3	0.1			0.2	0.1	0.1		1.2	tr			RI, MS
14	β-pinene	970	967	1110	0.1	0.1	0.1	1.7	0.2	0.3	0.4	5.3	6.5	tr	8	0.1	0.2	4.7	RI, MS

15	1,8-dehydro-cineole	979	977	1190		1													RI, MS
16	myrcene	976	980	1159	2.1	1.3	2.4	16.9	tr	1.2	1.3	1	0.8	tr	1.7		0.1	tr	RI, MS
17	3-Octanol	982	982	1350					0.8		0.2								RI, MS
18	α-phellandrene	997	998	1164	0.2		0.3					0.1	tr			tr	0.2	1.8	RI, MS
19	3-carene	1005	1006	1147	0.1		0.1					0.1	0.7	2.1	0.3				RI, MS
20	α-terpinene	1008	1010	1178	1.7	0.1	2.1	0.3			0.1	0.3	tr					2.8	RI, MS
21	p-cymene	1011	1013	1268	12.4	0.1	7.9	0.3			1	1	2	0.5		1.8	0.9	23.8	RI, MS
22	limonene	1020	1021	1206		4.1		5.6	1.1		0.3	1.2	tr			5.7	3.3		RI, MS
23	1,8-cineole	1020	1021	1215		2.9		6.5	0.6	3.8	0.2	49.4	9	14.8	67.7	69.9	80.2	42.5	RI, MS
24	β-phellandrene	1021	1021	1208			0.5					tr	0.7		2.1		0.2	0.1	RI, MS
25	Z-β-ocimene	1024	1026	1230	0.6	0.1	tr	0.1		0.4	0.2	0.1				tr	0.1		RI, MS
26	E-β-ocimene	1034	1037	1247		1.5	0.1	1.9		0.4	tr				tr	tr		1.5	RI, MS
27	γ-terpinene	1047	1049	1243	4.3	0.4	13.6	0.5	0.1	0.2	0.3	0.5	0.9	0.1	tr	0.1		2.4	RI, MS
28	*trans*-Hydrate sabinene	1051	1054	1444	0.1						3	0.1	tr		0.1				RI, MS
29	Fenchone	1071	1070	1401										37.8					RI, MS
30	p-cymenene	1075	1072	1430		0.1								tr					RI, MS
31	terpinolene	1078	1078	1280	0.2	0.5	0.2	0.4		0.1	0.5	0.2	0.4			tr	0.1		RI, MS

32	linalool	1081	1082	1544	1.7		0.8	2.1	tr	40.4		0.8	tr	0.7	tr		RI, MS
33	nonanal	1081	1082	1390				0.5									RI, MS
34	*cis*-sabinene hydrate	1083	1082	1541		0.3					0.1	tr	0.1				RI, MS
35	1-octen-3-yl acetate	1095	1092	1376		0.7			tr		0.1						RI, MS
36	perillene	1098	1095	1713				0.3									RI, MS
37	2-Methyl-butyl isovalerate	1098	1096	1274						0.4							RI, MS
38	Fencyl alcohol	1100	1099	1581										1.1			RI, MS
39	p-mentha-1,3,8-triene	1101	1103	1433		0.1											RI, MS
40	Cis-p-Menth-2-en-1-ol	1108	1110	1600						tr	0.1						RI, MS
41	3-Octyl acetate	1111	1110	1315						0.2							RI, MS
42	camphor	1123	1120	1517	0.1	1.4		25.9				13.2	23.5	19.5	3.2	tr	RI, MS
43	*trans*-pinocarveol	1125	1122	1650		0.4		0.6							tr	1.1	RI, MS
44	Trans-p-Menth-2-en-1-ol	1123	1126	1612						tr	tr						RI, MS
45	*cis*-verbenol	1127	1127	1650		0.5								0.3	0.1		RI, MS
46	trans-verbenol	1129	1130	1675										0.3			RI, MS
47	Menthone	1134	1135	1456					10.8		28.5						RI, MS
48	p-Menth-3-en-8-ol	1135	1135	1590							3.1						RI, MS

49	pinocarvone	1136	1137	1558		0.1		0.2				0.3	tr		0.2				RI, MS
50	Iso-Menthone	1143	1142	1490					0.7		19								RI, MS
51	Isoborneol	1143	1144	1670	0.5									0.5	0.9				RI, MS
52	borneol	1148	1149	1698	0.3	6.8	0.3	2.1			0.1	3	0.7			1.3			RI, MS
53	Neo-Menthol	1156	1157	1637					1.6		10.4								RI, MS
54	Cryptone	1157	1158	1667														5.9	RI, MS
55	terpinen-4-ol	1161	1160	1600	1.1	0.6	0.9	1.3			2.7	1.2	0.6	0.4	0.6	0.8	0.2	2.9	RI, MS
56	Menthol	1164	1163	1629					tr		1.4			0.9					RI, MS
57	Myrtenal	1172	1169	1628		0.1								0.7		tr			RI, MS
58	Iso-Menthol	1174	1173	1660					tr		2.1								RI, MS
59	α-terpineol	1179	1175	1700	0.1	9.7	0.1	1.1	tr	6.4	2.9	3.6	0.3		3.8	6.0	2.4	tr	RI, MS
60	Myrtenol	1177	1177	1789											0.2	0.1			RI, MS
61	α-Campholenol	1186	1188	1782							tr								RI, MS
62	*trans*-carveol	1196	1196	1832		0.2										0.1			RI, MS
63	Nerol	1211	1213	1799						1.1									RI, MS
64	Fenchyl acétate	1212	1213	1468										0.6					RI, MS
65	Pulegone	1213	1216	1640					77.3	0.1	5.6								RI, MS

66	cis-3-hexenyl-2-methyl butyrate	1220	1220	1457		tr										RI, MS
67	carvone	1222	1223	1739		0.1							tr	0.2	0.2	RI, MS
68	Piperitone	1232	1229	1727				0.3		1.3				0.1	0.2	RI, MS
69	Geraniol	1232	1234	1844					2.4						0.9	RI, MS
70	Linalyl acetate	1240	1237	1557				tr	32.6							RI, MS
71	Geranial	1244	1243	1731					0.2							RI, MS
72	Neryl formate	1263	1266	1647					0.1							RI, MS
73	thymol	1266	1267	2189	0.6		0.3								tr	RI, MS
74	Neo-Menthyl acetate	1263	1268	1548				0.1		5						RI, MS
75	bornyl acetate	1269	1268	1575		5.2					0.4	0.2	3.6		tr	RI, MS
76	Lavandulyl acetate	1270	1273	1593					0.1							RI, MS
77	carvacrol	1278	1280	2219	69.6		65.7								tr	RI, MS
78	Menthyl acetate	1282	1285	1578						2.1						RI, MS
79	Iso-Menthyl acetate	1294	1295	1594				0.1		1.8						RI, MS
80	Myrtenyl acetate	1301	1300	1680									3.7			RI, MS
81	Dihydro carvyl acetate	1311	1312	1661												RI, MS
82	Piperitenone	1315	1313	1900				2.7		1.8						RI, MS

83	thymyl acetate	1330	1328	1820			0.1												RI, MS
84	Eugenol	1330	1329	2164	0.1											0.1			RI, MS
85	α-terpinyl acetate	1336	1336	1681		51.7		0.2		0.1	0.1					1.1			RI, MS
86	carvacryl acetate	1346	1344	1874			0.2												RI, MS
87	Neryl acetate	1342	1345	1725						2.7									RI, MS
88	α-cubebene	1350	1347	1452				0.2						tr					RI, MS
89	Geranyl acetate	1361	1364	1725						2.5									RI, MS
90	α-copaene	1379	1376	1488		0.2		0.7			0.1	tr	0.6	0.6					RI, MS
91	β-bourbonene	1385	1382	1515		0.1		0.6		0.1	tr								RI, MS
92	β-elemene	1388	1386	1589		tr		0.3											RI, MS
93	isocaryophyllene	1407	1405	1571		tr													RI, MS
94	α-gurjumene	1413	1410	1524		0.1		0.1											RI, MS
95	E-β-caryophyllene	1420	1418	1591	1.6	0.5	1.8	2.2	0.3	0.8	0.4	0.7	1.9	0.2	0.5	tr	0.3	tr	RI, MS
96	E-β-farnesene	1448	1445	1661		tr		0.5		0.1	0.2	tr	0.1		0.1				RI, MS
97	α-humulene	1456	1453	1665	0.1		0.1		0.4			0.1	0.3		0.2				RI, MS
98	γ-muurolene	1471	1469	1681		0.3		3.4		0.1	0.2				tr				RI, MS
99	germacrene D	1480	1479	1692		0.1		0.7		0.1	0.1								RI, MS

100	β-selinene	1483	1487	1712				0.9											RI, MS
101	bicyclogermacrene	1492	1489	1727		0.1													RI, MS
102	α-Muurolene	1496	1492	1709							0.1								RI, MS
103	β-bisabolene	1500	1500	1720		0.7	0.1	0.5											RI, MS
104	γ-cadinene	1507	1505	1752		0.2		0.7	tr	0.2	0.2								RI, MS
105	trans-Calamenene	1512	1510	1810						0.2	0.1				0.3				RI, MS
106	δ-cadinene	1516	1514	1752		0.5		5.7											RI, MS
107	Cadina-1,4-diene	1523	1520	1763						0.1									RI, MS
108	α-Calacorene	1531	1528	1890							0.1								RI, MS
109	α-cadinene	1532	1530	1740				0.7	tr	0.2	0.1								RI, MS
110	E-α-bisabolene	1532	1532	1753			0.1												RI, MS
111	β-Calacorene	1548	1546	1936							tr								RI, MS
112	Spathulenol	1557	1556	2115												tr	tr	2.3	RI, MS
113	epi-globulol	1558	1556	2013										0.4					RI, MS
114	caryophyllene oxyde	1576	1572	1980	0.1	0.3		1.2		0.3		0.2	tr	0.2	0.2				RI, MS
115	Globulol	1580	1582	2074						0.5						0.1			RI, MS
116	Viridiflorol	1591	1590	2089										1.2		0.1			RI, MS

117	Tau-cadinol	1632	1634	2169		0.1													RI, MS
118	Cubenol	1632	1634	2013										0.6					RI, MS
119	Tau-muurolol	1634	1636	2143										0.2					RI, MS
120	α-bisabolol	1672	1669	2217		0.1		0.8							0.4				RI, MS
121	abietatriene	2042	2039	2493			0.2												RI, MS
122	abieta-7,13-diene	2080	2077	2457			0.1												RI, MS
	**Total Identification %**				**99.5**	**99.3**	**99.7**	**98.2**	**98.5**	**98.8**	**98.9**	**93.6**	**95.3**	**95.1**	**95.2**	**95.7**	**94.5**	**95.2**	
	**Hydrocarbon compounds**				**24.7**	**17**	**31.3**	**55.1**	**2.7**	**4.8**	**6.5**	**21.2**	**60.9**	**7.6**	**17.5**	**13.7**	**11.7**	**41.6**	
	Monoterpene hydrocarbons				23	14.2	28.9	37.9	2	2.8	4.9	20.4	58	6.8	16.4	13.7	11.4	41.6	
	Sesquiterpene hydrocarbons				1.7	2.8	2.1	17.2	0.7	2	1.6	0.8	2.9	0.8	1.1	tr	0.3	tr	
	Diterpene hydrocarbons					-	0.3	-											
	**Oxygenated compounds**				**74.8**	**82.3**	**68.4**	**43.1**	**95.8**	**94**	**92.4**	**72.4**	**34.4**	**87.5**	**77.7**	**82**	**82.8**	**53.6**	
	Oxygenated monoterpenes				74.2	81	68.4	40.3	94.2	92.5	91.3	72	34.4	84.9	77.1	81.8	82.8	51.3	
	Oxygenated sesquiterpenes				0.1	0.5	-	2	0	0.8	0	0.2	0	2.6	0.6	0.2	tr	2.3	

Non-terpenic oxygenated compounds	0.5	0.8	-	0.8	1.6	0.7	1.1	0.2	0	0	0	0	0	0

[a] Order of elution is given on apolar column (DB-5). Bold types refer to main compounds. [b] Retention indices of literature on the apolar column (lRIa) reported from [54,]. [c] Retention indices on the apolar DB-5 column (RIa). [d] Retention indices on the polar SPB-1 column (RIp)e. oils from each species and subspecies. Quantification was carried out using RFs relative to tridecane as internal standard. [%] Normalized percentages are given on the apolar column except for components with identical RIa (percentages are given on the polar column), tr = trace (< 0.05%). Sample oils: normalized percentages (%) from all sample oils of each subspecies are given on the apolar column except for components with identical RIa (percentages are given on the polar column), tr = trace (< 0.05%). The chemical compositions of all individual samples are tabulated in Supplementary Material. [f] RI: Retention Indice; MS: Mass Spectrometry in electronic impact mode. All compounds were identified by comparing their EI-MS and retention indices with references compiled in the in-house library.

Table S2. Yields of essential oil extractions of different species

Oil yield (%)	October	November	December	January	February	March	April	May	June	July	August	September
M. piperita	1.33± 0.02	0.85 ± 0.04	0.45±0.01	0.26± 0.22	0.57±0.12	0.62±0.00	0.38±0.05	0.65±0.01	0.98±0.02	1.02±0.04	1.28±0.13	1.82±0.02
M. rotundifolia	2.31± 0.03	1.72 ± 0.03	0.66±0.03	0.71±0.03	0.41±0.02	0.83±0.02	1.2±0.26	2.4±0.18	2.61±0.03	2.65±0.02	2.92±0.03	3.01±0.02
M. pulegium	1.69 ± 0.01	1.44 ± 0.02	1.2±0.35	0.29±0.05	0.71±0.03	0.65±0.03	0.42±0.04	0.93±0.03	1.2±0.33	1.32±0.14	1.45±0.03	1.75±0.03

Table S3. Percentages of major molecules

Compounds (%, normalized percentage)	October	November	December	January	February	March	April	May	June	July	August	September
Eucalyptol	32,7 ± 0,2	30,5 ± 0,26	25,9 ± 1,05	18,7 ± 0,76	16,9 ± 0,35	12,1 ± 0,44	6,7 ± 0,42	3,5 ± 0,25	2,1±0,17	1,9±0,1	0,8±0,02	28,2±0,29
Menthone	23,1 ± 0,2	27,2 ± 0,15	29,8 ± 0,55	30,5 ± 0,4	32,5 ± 2,11	34,2 ± 0,36	36,6 ± 0,35	40,5 ± 0,2	39,8±0,26	32,4 ± 0,3	29,6±0,26	25,1±0,17
Neo-menthol	19,8 ± 0,2	22,9± 0,9	32,5 ± 0,32	35,8 ± 0,26	25,5 ± 0,26	23,7±0,44	20,9 ± 0,17	14,8±0,42	10,3±0,3	9,8±0,35	7,3±0,0	15,4±0,36
Pulegone	5,9± 0,61	6,5± 0,36	9,1 ± 0,21	12,9 ± 0,4	15,8 ± 0,26	15,9 ± 0,09	17,9 ± 0,1	21,2±0,26	28,6±0,26	32,8±0,46	42,9±0,09	17,3±0,20

Table S4. Time-dependent variations

Compounds (%, normalized percentage)	October	November	December	January	February	March	April	May	June	July	August	September
Eucalyptol	3,7 ± 0,2	2,9 ± 0,17	1,5 ± 0,17	3,7 ± 0,36	5,1 ± 0,19	5,6 ± 0,26	6,4 ± 0,44	7,5 ± 0,46	4,3 ± 0,38	4 ± 0,00	3,9 ± 0,17	3,9 ± 0,15
Linalool	51,3 ± 0,69	50,9 ± 0,19	44,2 ± 0,12	40,2 ± 0,56	34,7 ± 0,70	30,9 ± 0,10	29,7 ± 0,10	39,6 ± 0,36	50,2 ± 0,2	52,1 ± 0,17	53,4 ± 0,5	59,4 ± 0,35
α-terpineol	8,9 ± 0,78	7,6 ± 0,4	7,1 ± 0,26	6,9 ± 0,1	2,7 ± 0,2	3,3 ± 0,4	5,1 ± 0,26	6,2 ± 0,2	8,1 ± 0,26	9,2 ± 0,26	10 ± 0,35	9,1 ± 0,26
Linalyl acetate	17,9 ± 0,10	18,2 ± 0,44	19 ± 0,02	20,1 ± 0,26	38,5 ± 0,26	39,2 ± 0,2	40,6 ± 0,61	35,2 ± 0,71	15,4 ± 0,2	14,1 ± 0,10	12,4 ± 0,3	17,3 ± 0,26

Table S5. GPS coordinates

	Thymus capitatus					
	**No**	**Voucher codes**	**Localities**	**GPS Coordinates**	**Yields**	**Altitude**
	S01	TCA-0944-KA1	Béni Snous 1	34°39'31''N ; 1°33'18''O	0.52	853
	S02	TCA-0944-KA2	Béni Snous 2	34°39'33''N ; 1°33'27''O	0.5	873
	S03	TCA-0944-KA3	Béni Snous 3	34°39'30''N ; 1°33'30''O	0.58	883
	S04	TCA-0944-KA4	Béni Snous 4	34°39'41''N ; 1°33'33''O	0.61	891
	S05	TCA-0944-KA5	Béni Snous 5	34°39'42''N ; 1°33'30''O	0.66	881
	S06	TCA-0944-KA6	Béni Snous 6	34°39'42''N ; 1°33'18''O	0.63	853
	S07	TCA-0944-KA7	Béni Snous 7	34°39'30''N ; 1°33'18''O	0.5	853
	S08	TCA-0944-KA8	Béni Snous 8	34°39'37''N ;1°33'20''O	0.73	856
	S09	TCA-0944-KA9	Béni Snous 9	34°39'46''N ; 1°33'35''O	0.66	893
	S10	TCA-0944-KA10	Béni Snous 10	34°39'30''N ;1°33'33''O	0.66	889
	S11	TCA-0944-KA11	Béni Snous 11	34°39'27''N ; 1°33'28''O	0.66	882
	S12	TCA-0944-KA12	Beni Hammou 1	34°38'40''N ; 1°32'08''O	0.52	797
	S13	TCA-0944-KA13	Beni Hammou 2	34°38'41''N ; 1°32'08''O	0.52	793
	S14	TCA-0944-KA14	Beni Hammou 3	34°38'49''N ; 1°32'40''O	0.54	790
	S15	TCA-0944-KA15	Beni Hammou 4	34°38'40''N ; 1°32'10''O	0.53	790
	S16	TCA-0944-KA16	Beni Hammou 5	34°38'45''N ; 1°32'13''O	0.69	770
	S17	TCA-0944-KA17	Beni Hammou 6	34°38'43''N ; 1°32'14''O	0.6	769

	Thymus munbyanus					
	**No**	**Voucher codes**	**Localities**	**GPS Coordinates**	**Yields**	**Altitude**
subsp. *munbyanus*	S1	TCM-0945-KA1	Boughedou 1	34°32'42''N ; 1°28'43''O	0.85	1164
	S2	TCM-0945-KA2	Boughedou 2	34°33'12''N ; 1°28'14''O	0.84	1140
	S3	TCM-0945-KA3	Boughedou 3	34°32'34''N ; 1°27'52''O	1.38	1173
	S4	TCM-0945-KA4	Ain Sfaa 1	34°31'20''N ; 1°29'55''O	1.22	1289
	S5	TCM-0945-KA5	Ain Sfaa 2	34°30'35''N ; 1°30'38''O	0.98	1367
	S6	TCM-0945-KA6	Sebdou 1	34°34'59''N ; 1°25'30''O	1.25	1040
	S7	TCM-0945-KA7	Sebdou 2	34°35'51''N ; 1°23'59''O	0.87	999
	S8	TCM-0945-KA8	Sebdou 3	34°34'09''N ; 1°26'49''O	0.96	1070
subsp. *eu-ciliatus*	S1	TCE-0945-KA1	koudia	34°54'38''N ; 1°20'57''O	0.61	628
	S2	TCE-0945-KA2	Sidi Weryach	35°11'10''N ; 1°30'31''O	0.66	216
	S3	TCE-0945-KA3	Souk El Khemis	35°10'19''N ; 1°34'11''O	0.69	214
	S4	TCE-0945-KA4	Boukiou	35°02'56''N ; 1°31'07''O	0.62	123
	S5	TCE-0945-KA5	Tounane	35°02'42''N ; 1°54'15''O	0.69	334
	S6	TCE-0945-KA6	Bab Teza	34°57'39''N ; 1°46'12''O	0.53	786
	S7	TCE-0945-KA7	Bourakba	34°55'19''N ; 1°45'41''O	0.55	503
	S8	TCE-0945-KA8	Ouled Riyad	34°57'05''N ; 1°30'03''O	0.49	297
	S9	TCE-0945-KA9	Ain Fezza	34°53'37''N ; 1°13'29''O	0.56	840
	S10	TCE-0945-KA10	Boughzel	34°47'48''N ; 1°29'00''O	0.76	907
	S11	TCE-0945-KA11	Tamekssalet	34°47'16''N ; 1°32'08''O	0.59	805
	S12	TCE-0945-KA12	Ain Ghoraba	34°43'07''N ; 1°23'26''O	0.63	879
	S13	TCE-0945-KA13	Sebdou	34°39'19''N ; 1°19'45''O	0.61	885
	S14	TCE-0945-KA14	Sidi Djillali1	34°27'03''N ; 1°34'26''O	0.96	1314
	S15	TCE-0945-KA15	El Abed	34°26'32''N ; 1°40'14''O	0.78	1230
	S16	TCE-0945-KA16	Sidi djilali 2	34°27'48''N ; 1°34'18''O	0.82	1514
	S17	TCE-0945-KA17	Sidi Djilali 3	34°27'21''N ; 1°35'21''O	0.79	1218
	S18	TCE-0945-KA18	Ouled abd slam 1	34°26'43''N ; 1°39'41''O	0.63	1261
	S19	TCE-0945-KA19	Ouled abd slam 2	34°27'20''N ; 1°39'36''O	0.41	1301
	S20	TCE-0945-KA20	El Bouihi 1	34°24'46''N ; 1°41'30''O	0.77	1248
	S21	TCE-0945-KA21	El Bouihi2	34°24'43''N ; 1°41'32''O	0.69	1247
	S22	TCE-0945-KA22	El buihi 3	34°24'45''N ; 1°41'45''O	0.77	1249
	S23	TCE-0945-KA23	Sidi lakhdar	34°23'56''N ; 1°40'51''O	0.43	1185
subsp. *coloratus*	S01	TCC-0945-KA1	Sebaa chioukh	35°09'27''N ; 1°20'40''O	0.33	524
	S02	TCC-0945-KA2	Ben Sekrane	35°04'23''N ; 1°11'58''O	0.37	418
	S03	TCC-0945-KA3	Hennaya	34°55'20''N ; 1°23'53''O	0.42	448
	S04	TCC-0945-KA4	Azayza	34°59'29''N ; 1°10'29''O	0.43	626
	S05	TCC-0945-KA5	Ain Isser	34°49'29''N ; 1°01'08''O	0.37	955
	S06	TCC-0945-KA6	sebdou	34°41'54''N ; 1°13'28''O	0.41	1039
	S07	TCC-0945-KA7	Nedrouma	35°00'33''N ; 1°43'13''O	0.35	604

	S08	TCC-0945-KA8	El Houanet	34°58'49''N ; 1°46'36''O	0.39	777
	S09	TCC-0945-KA9	Sabra	34°49'33''N ; 1°29'23''O	0.29	650
	S10	TCC-0945-KA10	Beni Snous1	34°38'54''N ; 1°33'23''O	0.58	887
	S11	TCC-0945-KA11	Tlemcen	34°51'29''N ; 1°21'17''O	0.51	909
	S12	TCC-0945-KA12	Beni Snous 2	34°38'43''N ; 1°30'45''O	0.45	731
	S13	TCC-0945-KA13	Beni snous 3	34°39'30''N ; 1°31'14''O	0.5	717
	S14	TCC-0945-KA14	Beni snous 4	34°39'36''N ; 1°30'49''O	0.41	712
	S15	TCC-0945-KA15	Beni snous 5	34°39'20''N ; 1°30'43''O	0.39	838
	S16	TCC-0945-KA16	Beni hammou1	34°38'33''N ; 1°32'21''O	0.51	777
	S17	TCC-0945-KA17	Beni hammou 2	34°38'40''N ; 1°32'13''O	0.42	780
	S18	TCC-0945-KA18	Khemis 1	34°38'36''N ; 1°33'51''O	0.33	867
	S19	TCC-0945-KA19	Khemis 2	34°38'35''N ; 1°33'51''O	0.21	868
	S20	TCC-0945-KA20	Khemis 3	34°38'22''N ; 1°33'34''O	0.44	842
	S21	TCC-0945-KA21	Khemis 4	34°38'26''N ; 1°33'28''O	0.39	844
	S22	TCC-0945-KA22	Ouled moussa 1	34°38'03''N ; 1°33'37''O	0.18	941
	S23	TCC-0945-KA23	Ouled moussa2	34°38'02''N ; 1°33'40''O	0.2	931
	S24	TCC-0945-KA24	Ouled moussa3	34°38'22''N ; 1°33'35''O	0.19	837
	S25	TCC-0945-KA25	Beni Bahdel 1	34°42'11''N ; 1°30'42''O	0.3	622
	S26	TCC-0945-KA26	Beni Bahdel 2	34°41'41''N ; 1°31'47''O	0.25	804
	S27	TCC-0945-KA27	Beni bahdel 3	34°41'31''N ; 1°32'21''O	0.23	917
	S28	TCC-0945-KA28	Bouhlou1	34°46'36''N ; 1°34'53''O	0.29	625
	S29	TCC-0945-KA29	Bouhlou2	34°46'32''N ; 1°34'54''O	0.26	651
	S30	TCC-0945-KA30	Bouhlou3	34°46'32''N ; 1°34'42''O	0.31	668
	S31	TCC-0945-KA31	Sid el abdli 1	35°03'16''N ; 1°08'58''O	0.11	450
	S32	TCC-0945-KA32	Sid el abdli 2	35°03'50''N ; 1°08'46''O	0.18	434
	S33	TCC-0945-KA33	Amieur 1	35°02'09''N ; 1°13'37''O	0.12	342
	S34	TCC-0945-KA34	Amieur2	35°01'33''N ; 1°13'34''O	0.15	390
	S35	TCC-0945-KA35	Ouled Mimoun	34°53'49''N ; 1°02'51''O	0.40	829
